# Yolk sac tumor in the abdominal wall of an 18-month-old girl: a case report

**DOI:** 10.1186/s13256-017-1216-4

**Published:** 2017-02-20

**Authors:** Machiel van den Akker, Dirk Vervloessem, An Huybrechs, Sabine Declercq, Jutte van der Werff ten Bosch

**Affiliations:** 1 Department of Pediatrics, ZNA Queen Paola Children’s Hospital, Antwerp, Belgium; 2 Department of Pediatric Surgery, ZNA Queen Paola Children’s Hospital, Antwerp, Belgium; 3 Department of Pediatrics, Heilig Hart Hospital, Lier, Belgium; 4 Department of Pathology, ZNA Middelheim Hospital, Antwerp, Belgium; 5 Department of Pediatric Hematology Oncology, UZ Brussel, Jette, Belgium

**Keywords:** Case report, Extragonadal germ cell tumor, Yolk sac tumor, Skin tumor, Children, Alpha-fetoprotein

## Abstract

**Background:**

Pediatric germ cell tumors account for approximately 3.5 % of all childhood cancers for children under the age of 15 years. Up to one-third are extragonadal neoplasms. Germ cell tumors are a heterogeneous group of malignant tumors with a wide variety of histopathological features. Yolk sac tumor is the predominant variant in newborns and younger children. We report for the first time, the presentation of a primary yolk sac tumor in the abdominal wall of a small child.

**Case presentation:**

An 18-month-old white girl underwent resection of a small, round subcutaneous lump (1.5×1.3×0.8 cm) of the abdominal wall in her right hypochondriac region. The histopathology was compatible with yolk sac tumor. Her alpha-fetoprotein was initially elevated but normalized after the resection. Magnetic resonance imaging of her abdomen was normal. The surgeon decided to observe and follow her alpha-fetoprotein level closely. One year after resection a local recurrence appeared and her alpha-fetoprotein rose to 58 ng/mL. The surgeon performed a wide resection of the lesion with normalization of her alpha-fetoprotein. Follow-up consisted of measuring alpha-fetoprotein, clinical evaluation, and abdominal ultrasound.

**Conclusions:**

Clinicians should be aware that a yolk sac tumor can present in an unusual extragonadal place, for example in this case it was subcutaneous. In some cases, conservative treatment can be carried out with careful monitoring of the patient and their alpha-fetoprotein.

## Background

Pediatric germ cell tumors (GCTs) account for approximately 3.5 % of all childhood cancers for children under the age of 15 years. Between the ages 15 and 19 the frequency goes up to 16 %. Up to one-third are extragonadal neoplasms and the most common sites are the sacrococcygeal or retroperitoneal region, and the pineal gland. The incidence of extragonadal tumors varies widely by age (higher in younger age) and gender (more often in girls at a younger age, while intracranial/intraspinal tumors are more common in boys at an older age) [1].

The only known risk factor for extragonadal GCTs is the presence of Klinefelter syndrome (47,XXY karyotype). In that case, there is a 50-fold increased risk of developing mediastinal GCTs in early adolescence [2, 3].

GCTs are a heterogeneous group of malignant tumors with a wide variety of histopathological features. Yolk sac tumor is the predominant variant in newborns and younger children, while later in life a wide range of histologic subtypes are seen. Yolk sac tumors have a microcystic reticular pattern and are cytokeratin-positive. Alpha-fetoprotein (αFP) expression is characteristic and can be used for diagnosis and monitoring of therapy. To the best of our knowledge, this is the first report of the presentation of a primary yolk sac tumor in the abdominal wall of a small child. After resection of the tumor, close monitoring was conducted, without any adjuvant chemotherapy.

## Case presentation

**Fig. 1:**
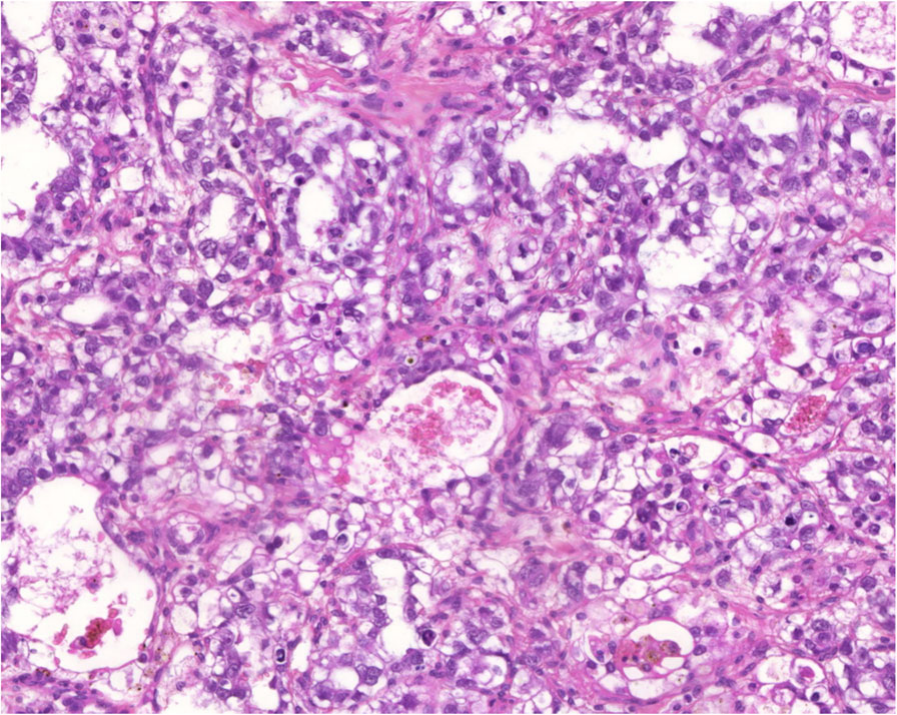
Yolk sac tumor with a reticular pattern formed by a loose meshwork of spaces (10×)

**Fig. 2:**
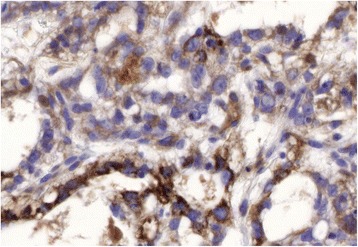
Yolk sac tumor with strong cytoplasmic positivity for alpha-fetoprotein

**Fig. 3:**
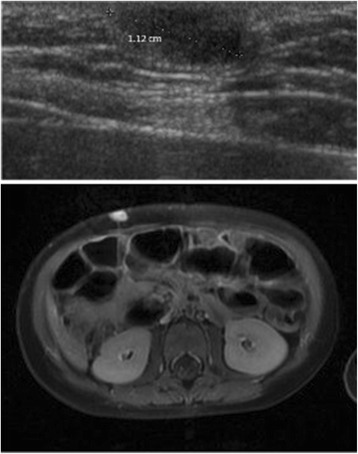
Ultrasound of the abdominal wall showing a subcutaneous oval-shaped nodule, hyporeflective, and without clear margins. Abdominal magnetic resonance imaging (axial oblique T1-weighted with contrast) with a contrast-captivating nodule in the right abdominal wall

**Fig. 4:**
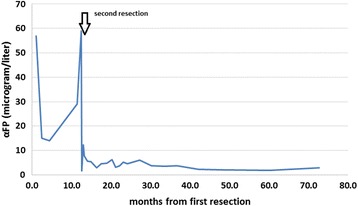
The course of alpha-fetoprotein in time (months) after the first resection. The *arrow* represents the second resection. *αFP* alpha-fetoprotein

An 18-month-old white girl underwent resection of a small, round subcutaneous lump (1.5×1.3×0.8 cm) of the abdominal wall in her right hypochondriac region. The tumor was connected to her skin and had the macroscopic appearance of a pilomatrixoma (epithelioma of Malherbe). Surprisingly the histopathology was compatible with yolk sac tumor, showing a microcystic reticular pattern (Fig. 1) with positive staining for cytokeratin 8 as well as cytoplasmic granular staining of αFP (Fig. 2). The resection borders were not completely clear from tumor tissue: stage II according to the Pediatric Oncology Group (POG)/Children’s Cancer Study Group (CCG) staging for malignant extragonadal GCT [4]. Her αFP was 57 ng/mL 3 weeks after resection and dropped to 15 ng/mL 1 month later; her beta subunit of human chorionic gonadotropin (ßHCG) was normal. An magnetic resonance imaging (MRI) of her abdomen was normal. The surgeon decided to observe and follow our patient’s αFP closely because of the lack of radiological evidence of the presence of a tumor and the decline in her αFP. Her αFP remained stable in the first months. One year after resection a local recurrence (1.1 cm, Fig. 3) appeared with an increase in her αFP to 29 ng/mL. An MRI of her head, neck, thorax, and abdomen did not show any other masses. The surgeon performed a wide resection of the lesion. The pathology report confirmed the previous findings; the borders were free of tumor tissue. Because the tumor was completely removed with normalization of her αFP (on the day of surgery her αFP was 58 ng/mL, 10 days later 12 ng/mL, 1 month postoperative 8 ng/mL) and there was no evidence of any other tumor masses, the management was expectative. The first year post-resection was uneventful without clinical signs of local recurrence and with normal monthly αFP level. An abdominal ultrasound, 1 year after resection, did not reveal a recurrence. Follow-up continued measuring αFP on a regular basis with gradually extending intervals (every 3 months in the second year, every 6 months in the third year and once a year in the fourth and fifth year). Clinical evaluation and abdominal ultrasound 5 years after the second resection showed a full tumor-free remission (Fig. 4). She was discharged from systematic follow-up.

## Discussion

GCTs consist of neoplastic cells arising from germ line cells (egg or sperm). They occur either in the testis or ovary, or outside the gonads. The extragonadal location is caused by either malignant transformation of aberrantly migrated primordial germ cell misplaced during the embryogenesis, or by a metastatic lesion of an undetected primary gonadal GCT not yet macroscopically visible or already spontaneously regressed. Extragonadal yolk sac tumors are rare and typically occur in midline locations, other sites have rarely been described [5]. Yolk sac tumors most often present in the first years of life and rarely with metastatic disease at diagnosis [6]. While age seems not to be a predictive factor, some authors describe the elevation of αFP to be prognostically important [7]. The αFP levels appear to correlate with the pathologic grade of retroperitoneal teratomas [8]. Without appropriate treatment, the tumor is highly aggressive, but with the combined treatment of surgery and adjuvant multi-agent platinum-based chemotherapy, a survival rate greater than 90 % can be achieved. The currently used regimens have comparable efficacy: PEI (cisplatin, etoposide, ifosfamide); carboPEI (carboplatin, PEI); BEP (bleomycin, etoposide, cisplatin); and carboplatin, etoposide, bleomycin [9].

Billmire *et al*. studied 25 children with malignant GCTs of the abdomen and retroperitoneum as the primary site and examined survival and event-free survival rates using high-dose or standard-dose cisplatin-based combination chemotherapy and surgical resection for these patients [10]. Most tumors were of advanced stage at diagnosis and in 15 patients histology showed pure yolk sac tumor. Of the 25 patients, four patients had their primary site located at the abdominal wall [10]. Maubec *et al*. reported an overview of primary skin GCTs, 16 of the 19 patients were children, and a mature teratoma was the most frequent diagnosis [11]. Tedgϋndϋz *et al*. described a 3-year-old girl with a subcutaneous paraspinal yolk sac tumor with metastatic disease located in the scar tissue at the surgical site and lumbar vertebrae. She received chemotherapy (cisplatin, etoposide, and bleomycin) and has been in remission for several years [12]. No other child with yolk sac tumor of the skin has been reported in the literature.

In yolk sac tumors, the tumor marker αFP is extremely sensitive for diagnosis and in follow-up after the appropriate treatment has been given [13]. αFP is an important serum binding protein in the fetus and is produced in the first trimester of fetal development by the yolk sac, afterwards by the fetal liver, and is gradually replaced by albumin. The αFP levels are usually highly elevated at birth with a significant variation in values among babies [14]. The half-life of αFP is approximately 5 to 6 days and normal adult levels (<10 IU/l) are achieved by the age of 2 years. Even then a wide variation in levels is observed [14] and some suggest that a mild elevation in αFP should not be used as the sole criterion to initiate or continue chemotherapy [15]. Therefore, in the first 2 years of life, αFP levels should be compared with age-related normal values. Serial measurements are necessary for optimal treatment decision. Most relapses occur within the first 2 years after diagnosis. Failure to normalize or a rise in the αFP level indicates a recurrence or incomplete resection of the yolk sac tumor [16], even before this can be shown by imaging methods.

αFP is regarded as a characteristic tumor marker of malignant GCTs and epithelial liver tumors. It is not tumor-specific. Elevated αFP in the serum of a child is also associated with benign conditions, for example hepatic disorders, hereditary disorders (for example ataxia telangiectasia, tyrosinemia type 1), systemic lupus erythematosus, and other malignant tumors (for example hepatoblastoma, hepatocellular carcinoma, pancreaticoblastoma, retinoblastoma) [17].

In regards of the patient presented, after the first resection, it was decided to observe her closely and not perform a wide resection. After local recurrence with an elevated αFP, a wide resection was done. No distant primary disease was found. Despite the availability of highly effective chemotherapy, we decided to observe. If the tumor reoccurs, then effective therapy can still be given. Due to the localization and the elevation of αFP we could easily perform intensive surveillance by monthly measurement of her αFP and yearly ultrasound of her abdomen (watch-and-wait strategy).

## Conclusions

We describe the first report of an 18-month-old girl diagnosed with a yolk sac tumor subcutaneously. A year after resection, she presented with local recurrence, which was treated with a wide resection only. Five years of follow-up did not reveal signs of local recurrence or distant disease. It is important that clinicians are aware that yolk sac tumor can present in an unusual extragonadal place. In some cases, conservative treatment can be carried out with careful monitoring of the patient and their αFP.
